# Interaction of E2 Glycoprotein with Heparan Sulfate Is Crucial for Cellular Infection of Sindbis Virus

**DOI:** 10.1371/journal.pone.0009656

**Published:** 2010-03-11

**Authors:** Wuyang Zhu, Lihua Wang, Yiliang Yang, Juan Jia, Shihong Fu, Yun Feng, Ying He, Jin-Ping Li, Guodong Liang

**Affiliations:** 1 Department of Viral Encephalitis, Institute for Viral Disease Control and Prevention, Chinese Center for Disease Control and Prevention (IVDC, China CDC) and State Key Laboratory for Infectious Disease Prevention and Control (SKLID), Beijing, China; 2 Department of Medical Biochemistry and Microbiology, University of Uppsala, Uppsala, Sweden; Institut Pasteur, France

## Abstract

Cell culture-adapted strains of Sindbis virus (SINV) initially attach to cells by the ability to interact with heparan sulfate (HS) through selective mutation for positively charged amino acid (aa) scattered in E2 glycoprotein (W. B. Klimstra, K. D. Ryman, and R. E. Johnston, J. Virol. 72: 7357–7366, 1998). Here we have further confirmed that interaction of E2 protein with HS is crucial for cellular infection of SINV based on the reverse genetic system of XJ-160 virus, a Sindbis-like virus (SINLV). Both SINV YN87448 and SINLV XJ-160 displayed similar infectivity on BHK-21, Vero, or C6/36 cells, but XJ-160 failed to infect mouse embryonic fibroblast (MEF) cells. The molecular mechanisms underlying the selective infectivity of XJ-160 were approached by substituting the E1, E2, or both genes of XJ-160 with that of YN87448, and the chimeric virus was denominated as XJ-160/E1, XJ-160/E2, or XJ-160/E1E2, respectively. In contrast to the parental XJ-160, all chimeric viruses became infectious to wild-type MEF cells (MEF-*wt*). While MEF-*Ext* ^−/−^ cells, producing shortened HS chains, were resistant not only to XJ-160, but also to YN87448 as well as the chimeric viruses, indicating that the inability of XJ-160 to infect MEF-*wt* cells likely due to its incompetent discrimination of cellular HS. Treatment with heparin or HS-degrading enzyme resulted in a substantial decrease in plaque formation by YN87448, XJ-160/E2, and XJ-160/E1E2, but had marginal effect on XJ-160 and XJ-160/E1, suggesting that E2 glycoprotein from YN87448 plays a more important role than does E1 in mediating cellular HS-related cell infection. In addition, the peptide containing 145–150 aa from E2 gene of YN87448 specifically bound to heparin, while the corresponding peptide from the E2 gene of XJ-160 essentially showed no binding to heparin. As a new dataset, these results clearly confirm an essential role of E2 glycoprotein, especially the domain of 145–150 aa, in SINV cellular infection through the interaction with HS.

## Introduction

Sindbis viruses are enveloped, single-strand RNA viruses, belonging to the genus of *Alphavirus* in the family *Togaviridae* that has more than 30 members [1]. Some of the members of the *Alphavirus* genus, such as Venezuelan, Eastern, and Western equine encephalitis viruses (VEEV, EEEV, and WEEV, respectively), can cause fever and viral encephalitis in human beings, resulting in mass epidemics or outbreaks exampled by the occurrences in South America and North America [2]–[4]. Due to the relative difficulty and risk of handling these dangerous pathogenic alphaviruses, Sindbis virus (SINV), that normally causes a mild rash and arthritis in humans but can cause fatal encephalomyelitis in mice, has been used extensively as a model system for the study of the infectivity and pathogenesis of *Alphavirus*.

The cell surface receptors for SINV to infect a broad variety of species have not yet been conclusively determined, but it has recently been shown that cell surface heparan sulfate (HS) found in both vertebrate and invertebrate species is involved in SINV infection and pathogenisis [5], [6]. Other wild-type alphaviruses, such as VEEV, Semliki Forest virus (SFV) and Ross River virus (RRV), were also shown HS-binding phenotype of infection during tissue culture [5], [7], [8]. In addition, HS has been shown to serve as a co-receptor for a number of viral infections, including herpes simplex virus [9], human immunodeficiency virus type 1 (HIV-1) [10], adeno-associated virus type 2 [11], respiratory syncytial virus [12], foot-and-mouth disease virus (FMDV) [13], and human papillomavirus type 11 [14] and so on. HS is a negatively chargedd linear carbohydrate polymer composed of repeating disaccharide of glucosamine and hexuronic acids that are sulfated at various positions [15]. Apart from the essential functions in animal development and homeostasis, demonstrated by targeted disruption of the enzymes involved in biosynthesis of HS [16], [17], HS is drawing attention as a potential target for prevention of viral infection. Previous investigations on the SINV-HS interaction indicate that HS-dependent phenotype has a selective advantage through the adaptive mutation for positively charged animo acid (aa) during tissue culture. Generally, non-tissue culture adapted SINV infect host cells by a HS-independent mechanism, while several of adaptive mutations for positively charged aa scattered in E2 gene have been found to increase viral infectivity by enhancing the binding or attachment to HS on the surface of the cell surface [5], [18], [19]. In addition, HS-binding phenotype could have implications for SINV virulence [19]–[22].

Based on the divergence of nucleotide sequencing and biological characteristics, Sindbis virus can be divided into two groups: SINV and Sindbis-like virus (SINLV) [23]. XJ-160 virus (GenBank No. AF103728) was isolated from a pool of *Anopheles* mosquitoes collected in Xinjiang, China [24]. The overall genomic sequence of XJ-160 has diverged significantly from the prototype AR339 with an 18% difference in nucleotides and an 8.6% difference in aa sequence. Notably, XJ-160 has displayed a biased reciprocal neutralizing reaction with SINV. XJ-160 can be efficiently neutralized by antiserum to SINV, while there is almost no neutralizing effect on SINV by antiserum to XJ-160. Therefore, XJ-160 was accordingly characterized as a SINLV [24]. In contrast, YN87448 virus (GenBank No. AF103734) was isolated from a female patient with fever in Yunnan Province, China, and differed less than 1% in nucleotide sequence from AR339. YN87448 virus can be efficiently neutralized by antiserum against SINV and was accordingly identified as a SINV [25]. Both XJ-160 and YN87448 can effectively replicate and cause a typical cytopathic effect (CPE) in BHK-21, Vero, or C6/36 cells [24], [25].

In this study, we have confirmed that interaction of E2 protein with HS is crucial for cellular infection of SINV. YN87448 virus was found to cause typical CPE in mouse embryonic fibroblast (MEF) cells, while XJ-160 virus failed to reproduce in this cell line. The molecular mechanisms of XJ-160's selective infection were investigated by substituting E1, E2, or both genes of XJ-160 virus with those of the YN87448 virus. Subsequently we compared the infectivity of parental with that of the chimeric viruses in wild-type (MEF-*wt*) and HS defect MEF (MEF-*Ext* ^−/−^) cells and their sensitivity towards treatment with exogenous heparin or heparinase I. The results demonstrated that E2 glycoprotein from YN87448 can help XJ-160 to overcome the resistance to infection by MEF cells through interaction with HS. In addition, specific interaction of E2 peptide from YN87448 with heparin further suggests that a domain of 145–150 aa from the E2 gene may be a molecular basis for the specific interaction of SINV with cellular HS.

## Results

### Infectivity and growth capacity of YN87448 and XJ-160 in BHK-21 cells

**Figure 1:**
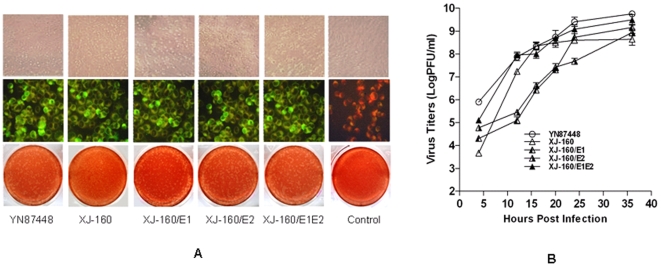
Production and growth properties of viruses in BHK-21 cells. (A) CPE 36 h postinfection (p.i.) (*upper panels*), IFA 48 h p.i. (*middle panels*), and plaque-forming assay 24 h p.i. (*lower panels*) of chimeric viruses and parental virus in BHK-21 cells. (B) Growth curves of chimeric viruses and the parental viruses. A monolayer of BHK-21 cells at 80% confluence was infected with parental viruses and recombinant viruses at a multiplicity of infection (MOI) of 0.01. The medium (1 ml) was removed at hours 4, 12, 16, 20, 24, and 36 p.i., and frozen for later determination of virus titers, and an equal volume of fresh medium was added. The virus titers are shown as the mean ± SD of three independent replicate experiments.

Inoculation of YN87448 and XJ-160 to BHK-21 cells resulted in positive immunofluorescent staining with antibodies that recognize virus specific protein. By contrast, the control dishes were negative for the immunostaining. (Fig. 1A). Both viruses caused a similar CPE in BHK-21 cells, characterized by rounding, aggregation and fusion properties. Both viruses formed clear plaques with similar morphological characteristics, although the time course of plaque formation by YN87448 was shorter and the plaque diameters formed by YN87448 were larger than those formed by XJ-160. The two viruses had a similar growth curve in BHK-21 cells, with an indistinguishable growth tendency. They exhibited rapid propagation from 4 h to 20 h after inoculation, and that peaked at 36 h after inoculation (Fig. 1B). In addition, our earlier studies showed that both YN87448 and XJ-160 could efficiently infect Vero and C6/36 cells in a similar manner [24]. These findings demonstrated that both SINV YN87448 and SINLV XJ-160 exhibited a similar infectivity and growth capacity in either mammalian cells or mosquito cells, such as BHK-21, Vero, and C6/36 cells.

### Generation of chimeric viruses and characterization of their infective properties

**Figure 2:**
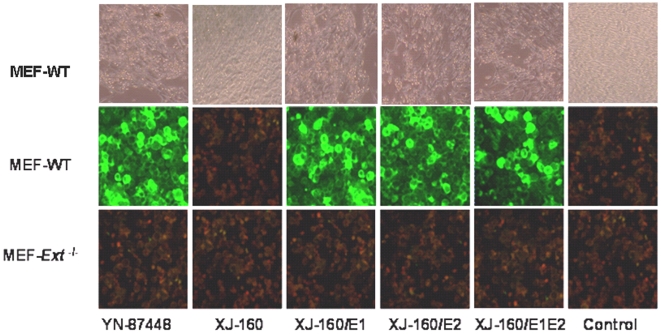
CPE 36 h p.i. on MEF-wt cells (*upper panels*), IFA 48 h p.i. on MEF-wt cells (*middle panels*) and IFA 48 h p.i. on MEF-*Ext* ^−/−^cells (*below panels*). MEF cells were grown on coverslips to 80% confluence and infected with YN87448 and XJ-160 as well as their chimeric products for 48 h. Antisera against YN87448 (used in YN87448 and for each of the chimeric viruses) or XJ-160 (used in the XJ-160 panel) diluted 1:100 were applied for IFA as described in the Materials and Methods. Uninfected MEF cells were used as controls.

Although SINV YN84448 and SINLV XJ-160 exhibited a similar characteristic in infecting BHK-21, Vero and C6/36 cells, they displayed a distinct phenotype in their infection of a mouse embryonic fibroblast cell line (MEF-*wt*). YN87448 effectively replicated and caused typical CPE in MEF-*wt* cells, while XJ-160 failed to reproduce in this cell line (Fig. 2). To discover the molecular basis for the differential infectivity of these two viruses toward different cell lines, three chimeric XJ-160/YN87448 viruses, XJ-160/E1, XJ-160/E2 and XJ-160/E1E2, were generated by replacing E1, E2, or both genes of XJ-160 with the corresponding genes from YN87448, using an *in vitro* transcription and electroporation method as described in the Materials and Methods. The whole genome in each chimeric virus was then sequenced, and the results confirmed the absence of mutations with the exception of the engineered recombination in these virus stocks.

As shown in Fig. 1A, all chimeric viruses caused plaque formation in BHK-21 cells, with a similar phenotype to that caused by YN87448, e.g., consistent in size (about 1 mm diameter) and shape (sub-circular), demonstrating that the successful recombinant constructions of the E1 and E2 genes that were competent in expression of the glycoproteins and assembling of the virions. It should be pointed out that the chimeric viruses caused plaque formation, on average, 12 h earlier and resulted in larger plaques than the parental XJ-160 virus did. Determination of the titers confirmed that the chimeric viruses released almost equivalent number of infectious particles as their parental viruses (Fig. 1B). Thus, it is clear that the E1 and E2 glycoprotein contributed to the selective infectivity of XJ-160 virus.

### Viral infectivity and reproductive properties in MEF cells

It has been reported that some SINV strains, such as AR332 after adaptation in culture, have selected HS as an attachment receptor through binding to the polysaccharide [6], [7]. To investigate whether YN87448 and XJ-160 as well as the chimeric viruses also require cell surface HS for infection and reproduction, we compared the infectivity of the viruses in MEF-*wt* with that in a HS mutant MEF cell line. MEF-*Ext* ^−/−^ cells are derived from mice that are deficient in one of the HS polymerases, EXT1 [26]. Characterization of the MEF cells generated from the mutant embryos illustrated that the MEF-*Ext* ^−/−^ expressed abnormal HS has a significantly shorter chain length (20 kDa), in comparison with the wild-type HS (70 kDa) [27].

**Figure 3:**
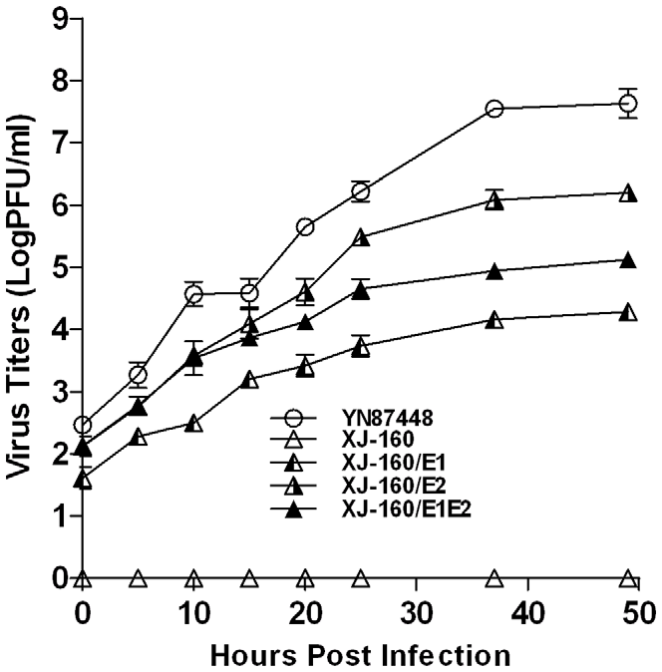
Growth curves of chimeric viruses on MEF cells. Monolayers of MEF-*wt* were infected with chimeric or parental viruses at a MOI of 0.01, and the medium (1 ml) was removed at the indicated time points and evaluated for virus titer by plaque assays. Each point represents the mean ± SD of three wells.

XJ-160 virus failed to infect any of the MEF cell lines, as demonstrated by IFA (Fig. 2), while YN87448, XJ-160/E1, XJ-160/E2 and XJ-160/E1E2 caused infection of MEF-*wt* cells with an apparently similar pattern (Fig. 2). Quantification of the virus particles in culture medium revealed different kinetics of viral reproduction (Fig. 3A). YN87448 showed the highest reproductive ability, followed by XJ-160/E2 and XJ-160/E1E2. XJ-160/E1 had the lowest titer 50 h post-infection among YN87448 and all chimeric viruses. MEF-*Ext* ^−/−^ cells were resistant to all viruses, likely due to the shorter HS chains expressed on their cell surface. These results indicate that the inability of XJ-160 to infect MEF cells was related with its incompetent discrimination of cellular HS.

### Inhibition of plaque formation by heparin or heparinase I treatment

**Figure 4:**
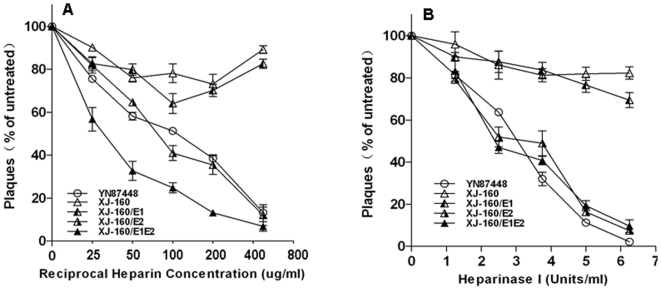
Effect of heparin or heparinase I treatment on plaque formation by the chimeric or parental viruses. (A) Viruses diluted to 100–200 PFU/200 µl were incubated with heparin at the concentrations indicated for 1 h at 37°C. Then plaque assays were performed using BHK-21 cells as described in the Materials and Methods. (B) Confluent BHK-21 cell monolayers were treated with heparinase I at the concentrations indicated. After washing three times with PBS, the cells were infected with viruses diluted to 100–200 PFU in 200 µl. Plaque formation was analyzed as described.

Heparin, an analog of HS, has been reported to be able to block SINV to infect and form plaques in cells [5]. It is of interest to know whether the infection of XJ-160 and YN87448 viruses and their chimeric products can also be affected by heparin Pre-incubation of heparin with the viruses resulted in a dose-dependent inhibition of viral infection in BHK-21 cells (Fig. 4A). Heparin effectively blocked infectivity of YN87448, XJ-160/E2, and XJ-160E1/E2 viruses, with a 50% inhibition at 30–100 µg/ml and a 90% inhibition at 500 µg/ml. In comparison, the maximum inhibition of heparin for XJ-160 and XJ-160/E1 was about 30%.

Heparinase I specifically cleaves heparin and HS, and has been used to remove cell surface HS for various biological studies [28]. We treated BHK-21 cells with this enzyme and evaluated the infectivity of the parental and chimeric viruses. Although the treatment resulted in a reduction of plaque formation in all viral infections in a dose-dependent manner, the inhibitory effect varies dramatically for different viruses. The infectivity of YN87448, XJ-160/E2, or XJ-160/E1E2 was essentially abolished after heparinase I treatment at higher concentrations, while the infection of XJ-160 and XJ-160/E1 was inhibited to a marginal degree only (around 20%) (Fig. 4B). These data confirm that E2 glycoprotein plays a bigger role in mediating cellular infection than E1 glycoprotein does.

### Interaction of heparin with peptides from E2 glycoprotein of XJ-160 and YN87448

**Figure 5:**
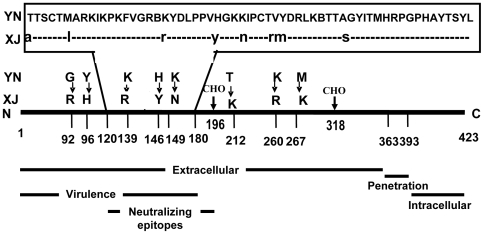
Alignment E2 glycoprotein gene of XJ-160 virus and YN87448 virus and linear map of E2 glycoprotein. CHO: carbohydrate attachment site.

**Figure 6:**
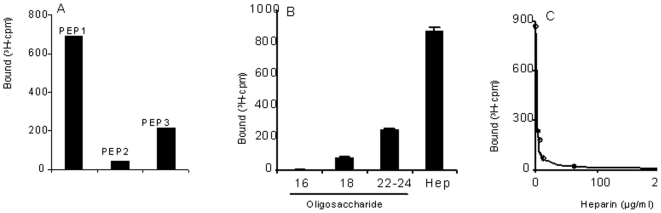
Binding of E2 peptides with heparin and heparin fragment. (A) The peptides (5 µg) were incubated with 10,000 cpm of ^3^H-labeled heparin at room temperature for 2 h; the incubated mixtures were analyzed by a filter-binding assay as described in the Materials and Methods. The bound ^3^H-labeled heparin was measured by scintillation counting. (B) Interaction of PEP1 (5 µg) with ^3^H-labeled heparin or heparin-derived oligosaccharides (16, 18, 22–24 mers). The data are presented as mean ± SD of three independent experiments. (C) PEP1 (5 µg) was incubated with 10,000 cpm of ^3^H-labeled heparin in the presence of unlabeled heparin at the concentrations indicated. The bound ^3^H-labeled heparin was measured by scintillation counting.

The E2 sequence of Sindbis virus can be divided into extracellular domain (1–363 aa), penetration domain (364–392 aa) and intracellular tail region (393–423 aa), among which the extracellular domain is a major receptor-binding region important for virus entry and infectivity. This region is postulated to constitute two domains conforming to the XBBXBX and XBBBXXBBX (B, basic; X, any amino acid) heparin-interaction consensus motifs identified by Cardin and Weintraub [29]. As shown in Fig. 5, one potential HS-binding motif locates in the sequence (127–132 aa) of both XJ-160 and YN87448; the other HS-binding site is found in the sequence (145–150 aa) of E2 glycoprotein. To further illustrate the molecular basis for the difference in the viruses with regard to response to heparin inhibition and heparinase I treatment, we produced peptides from E2 glycoprotein of YN87448 and XJ-160 as described in the Materials and Methods. Interactions of the peptides with radioisotope labeled heparin and its fragments were assessed by a nitrocellulose-filter trapping method [30]. Both PEP1 and PEP3 (129–150 aa and 137–169 aa of YN87448, respectively) bound to heparin (Fig. 6A), albeit that PEP1 showed a higher binding capacity than PEP3. PEP2 (129–150 aa of XJ-160) essentially showed no binding to heparin. The interaction of PEP1 with heparin is size-dependent, as heparin fragments smaller than 18-mers were unable to interact with the peptide (Fig. 6B). To verify the specificity of the interaction, unlabeled heparin was included in the incubation to compete with the ^3^H-labeled heparin in the reaction. Complete inhibition of ^3^H-labeled heparin binding to the peptide indicated that the heparin indeed specifically bound to the E2 peptide (Fig. 6C). These results indicate that the HS-binding domain of 145–150 aa in the E2 gene might be a molecular basis by which YN87448 can confer the ability of XJ-160 to recognize cellular HS.

### Neurovirulence of the chimeric viruses *in vivo*

**Figure 7:**
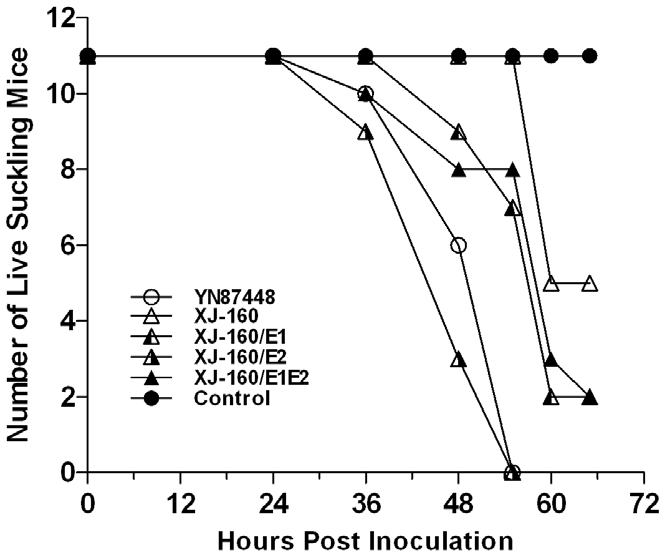
Neurovirulence of the viruses for suckling mice. Suckling mice (3 days old) were inoculated intracerebrally with 30 µl of 10^3^ PFU/ml chimeric or parental viruses; an equal volume of Eagle's medium supplemented with 1% fetal bovine serum was used in control mice.

To investigate the effects of the glycoprotein substitution on neurovirulence of XJ-160 virus, we inoculated suckling mice (BALB/c) intracortically (i.c.) with the chimeric and their parental viruses. All the viruses showed fatal neurovirulence in suckling mice; however, the survival rate and lethal time varied. YN87448 and XJ-160/E2 viruses caused animal death 36 h p.i., and killed all animals about 2 days after inoculation. In comparison, XJ-160 inoculation did not result in any animal death until 60 h p.i. The chimeric viruses XJ-160/E1 and XJ-160E1/E2 exhibited moderate degree in neurovirulence, showing stronger virulence than the parental virus XJ-160, but significantly weaker virulence than YN87448 and XJ-160/E2 (Fig. 7). These results, again, suggest that the E2 glycoprotein plays critical role for neurovirulence of the SINV in suckling mouse, which is correlated to HS-mediated cell entry.

## Discussion

Adaptive mutations for positively charged aa, such as E2-1 Ser–Arg, E2-70 Glu–Lys, or E2-114 Ser–Arg, were found to enhance binding and infectivity by allowing SINV to attach the cells via a HS-dependent mechanism [5], [18], [19]. Similar to this, adaptive mutations increasing infectivity of other wild-type alphaviruses, such as RRV and SFV, were also found to locate in the E2 gene [5], [8], [18]. The cell surface binding of RRV (T48 strain) was previously found to be independent of HS, while SINV (Toto 1101 strain) utilizes HS as a co-receptor to infect cells [6], suggesting that the contributions of cellular HS to host infection varies with different alphaviruses. However, repeated passage of RRV in chicken embryo fibroblast (CEF) cells where the virus titer declined during initial passages, but increased from the fifth passage. Then it was demonstrated that adaptive mutation of Asn to Arg or Lys at E2 (218 aa) created a HS-binding site [8], which may have contributed to the increased infectivity. Notably, all adaptive mutations for positively charged aa were found to scatter in E2 gene. In line with previous findings, our present investigations have demonstrated that E2 glycoprotein of SINV plays a more important role than E1 glycoprotein in mediating HS-dependent cellular infection. This is also agreed with the early reports that E2 glycoprotein contains more potent antigenic epitope clusters and receptor-binding sites than does E1 glycoprotein, while E1 glycoprotein in the center of the spike structure is coated by E2 glycoprotein [31], [32].

E2 glycoprotein from mature SINV particles have been postulated to constitute two heparin-binding domains located at 127–132 aa and 145–150 aa, respectively [29]. Analyses of amino acid indicate that electrostatic changes of E2 glycoprotein among Sindbis viruses mostly occur in the second HS-binding region located in the sequence of 145–150 aa, while few electrostatic changes were found in the first HS-binding domain located in the region of 127–132 aa (ARKIKP) (data not shown). From our data of heparin-E2 peptide interaction (Fig. 4) we speculate that the binding sites of YN87448 to HS are composed of two parts, e.g. the linear HS-binding domains similar to the XBBXBX or XBBBXXBBX motifs and the scattered positively chargedd amino acids located in the E2 glycoprotein. For example, alignment of the E2 glycoprotein sequences from YN87448 and XJ-160 revealed identical sequences at the first heparin-binding domains, but differences at the other domain where the two positively chargedd aa (His and Arg at 146 and 149, respectively) of SINV YN87448 are changed to two neutral amino acids in SINLV XJ-160 (Fig. 5). This may explain that SINLV XJ-160 is not HS-dependent in infection of cells due to lacking of the two basic loci in the second HS-binding domain. In comparison, E2 glycoprotein of YN87448 has the two potential HS- binding sites, accordingly can bind to cellular HS as previously [5]–[8]. These observations, along with the previous findings, suggest that Sindbis virus may be divided into two groups, a HS-dependent group and a HS-independent group, based on the electrostatic characteristics of the second HS-binding region in E2 glycoprotein. The HS-dependent group includes all Sindbis viruses containing the second HS-binding region (145–150 aa) characterized as XBXBBX; while Sindbis viruses containing two neutral amino acids at 146 and 149 of E2 glycoprotein are classified as the HS-independent group. According to this, SINV AR339 (GenBank No. NC_001547), YN87448 (GenBank No. AF103734), and Gird Wood (GenBank No. U38304) strains belong to the HS-dependent group, while the SINLV SW6562 (GenBank No. AF429428), Kyzylagach (GenBank No. AF023291), and XJ-160 (GenBank No. AF103728) strains should possess an HS-independent phenotype.

It is worth noting that there are two carbohydrate attachment sites (CHO) in E2 of SINV, being located at E2-196 and E2-318 [33]. Experimental data showed that eliminating either of the carbohydrate attachment sites increased SINV binding to heparin [34]. In this study, we compared E2 glycoprotein from SINLV XJ-160 and SINV YN87448, and found that both viruses have the carbohydrate attachment sites in E2-196 and E2-318 (Fig. 5), suggesting that the glycosylation is not an essential genetic determinant for the differential HS-dependent phenotype of the two viruses.

It is well established that HS is also involved in pathological processes by mediating infection of diverse microbial entities including viruses. The most direct evidence is obtained through studies of infection by herpes simplex viruses that require a specific fine structure of HS for interactions [35]. Other evidence suggests that effect of the affinity of alphaviruses for HS on viral pathogenesis is correlated to the manner of inoculation. Several descendant viruses from AR339 with single HS-binding mutations that attenuatedshowed low virulence after subcutaneous (s.c.) inoculation, but high virulence when inoculated directly into the brain of mice [19]–[22]. Consistent with this, chimeric XJ-160/E2 with an HS-dependent characteristic had a higher pathogenic capacity in neonatal mice than the parental virus XJ-160 when inoculated i.c. (Fig. 7). These results suggest that HS binding may attenuate viral infection that is dependent on high-titer viremia, however, efficient cell attachment through the interaction with HS can increase virulence, possibly through enhancing the replication of SINV within specific host tissues such as brain.

Taken together, we have characterized two virus strains, SINV YN87448 and SINLV XJ-160, with regard to the correlation between viral infection and cellular HS. As a new dataset.E2 glycoprotein was confirmed to play an essential role in SINV cellular infection through interaction with HS. Our results also suggest that the second HS-binding domain (145–150 aa) in E2 glycoprotein maybe the genetic determinant of Sindbis virus discriminating cellular HS, Accordingly, SINV is proposed to be divided into HS-dependent group and HS-independent group based on the structure of E2 protein.

## Materials and Methods

### Ethic statements

#### Approval Notice

The Ethic Review Committee of Institute for Viral Disease Control and Prevention, China CDC has reviewed the proposed use of experimental animals in the project entitled “Heparan Sulfate-independent Infection of Mammalian Cells by Sindbis-like Virus XJ-160”. It is recognized that the right, the husbandry, the experimentation and the welfare of the subjects are adequately protected, and under the supervision of the Committee.

Institute for Viral Disease Control and Prevention, China CDC

Ethic Review Committee

DATE: May 27, 2009

### Virus strains and cell culture

Both XJ-160 virus (GenBank No. AF103728) and YN87448 virus (GenBank No. AF103734) were isolated in China and are stored in our laboratory. Baby hamster kidney (BHK-21) cells were maintained in Dulbecco's Modified Eagle's Medium (DMEM) supplemented with 10% fetal bovine serum (FBS) and 100 U/ml each of penicillin and streptomycin [36], [37]. XJ-160 and YN87448 have been passaged about 50–60 times in BHK-21 cells in our lab. Mouse embryonic fibroblast (MEF) cells were generated from mouse embryos that are mutated in HS biosynthesis and the properties of the mutant strains, MEF-*Ext* ^−/−^, were characterized as described [27]. The cells were cultured in DMEM supplemented with 10% FBS and 100 U/ml each of penicillin and streptomycin.

### Production and identification of the chimeric viruses

Based on the backbone of full-length XJ-160 cDNA clone pBR-XJ160 [38], three recombinant XJ-160/YN87448 cDNA clones, designated pBR-XJ-160/E1, pBR-XJ-160/E2 and pBR-XJ-160/E1E2, respectively, were constructed using Platinum Pfx DNA Polymerase System kits (Invitrogen, USA). The glycoprotein genes E1, E2, or both of XJ-160 were replaced with those of YN87448 in the constructs. RNA transcripts were obtained by *in vitro* transcription of three recombinant clones using a mMESSAGE mMACHINE transcription SP6 kit (Ambion, UK). After capping and purification, the transcripts were electroporated into BHK-21 cells using Gene Pulser Xcell apparatus (Bio-Rad, USA) at 160 V, 25 F, and 200 Ω. After electroporation, the BHK-21 cells were incubated on ice for 10 min, then DMEM supplemented with 10% FBS was added. When the cell monolayer grew to confluence (after about 24 h), the culture medium was replaced with maintenance medium (DMEM supplemented with 2% FBS). At 48 h postinfection, the culture supernatants were collected to infect another monolayer of BHK-21 cells. The cytopathic effect (CPE) was examined daily using light microscopy. Finally, all chimeric viruses were confirmed and identified by sequencing analysis (Shanghai Shenggong), and the supernatants collected from BHK-21 cells were stored at −80°C.

### Indirect immunofluorescence assay

To detect viruses in the infected cells, an immunofluorescence assay (IFA) was performed as described [36]. In brief, the cells were seeded onto a glass coverslip in 35 mm diameter dishes. At 80% confluence, the cells were transfected with either transcript RNAs or the culture supernatant from the cells infected with the viruses. The cells were fixed in cold acetone when the CPE achieved “+++” (about 75% dead cells). After open-air drying and a second wash with cold phosphate buffered saline (PBS), 1∶100 diluted anti-XJ-160 or anti-YN87448 antibodies prepared in our lab were added and the combination incubated at 37°C for 30 min in a moist chamber. After washing three times with PBS and air drying, a second antibody (FITC-labeled sheep anti mouse antibody) diluted with azovan blue to 1∶100 was added to each well and incubated at 37°C for 30 min. Finally, the slides were washed three times with PBS, mounted with 90% glycerin, and observed under a fluorescence microscope.

### Plaque assay

Observation of plaque morphology and the determination of virus titer were performed by plaque assay as described earlier [37]. Briefly, the cells were grown to confluence in a 6-well plate to which 0.5 ml of diluted virus sample was added. After 1 h incubation at 37°C, the supernatant was removed, followed by adding 2 ml of culture cover medium (2×DMEM containing 2% FBS equivalently mixed with 2% ultraPure agarose). Then, the plate was turned over and incubated at 37°C in a humidified atmosphere with 5% CO_2_. When the CPE appeared under microscopic examination, another 2 ml culture cover medium containing 10% neutral red (Sigma, USA) was added and the plates were incubated at 37°C under 5% CO_2_ for 6 h. The size and number of plaques were then analyzed and recorded.

### Titer determination

Growth curves of the viruses were established in BHK-21 and MEF cells. The cells were grown to confluence in 75 cm^2^ flasks and infected at a multiplicity of infection (MOI) of approximately 0.01 plaque-forming units (PFU)/cell. After adsorption at 37°C for 1 h, 30 ml of maintenance DMEM containing 2% FBS was added and the cultures were incubated at 37°C under 5% CO_2_. Aliquots of culture medium were removed at different times as indicated and the titers of the virus released into the medium during each interval were determined by virus plaque assay. The results shown in this study are the means of three independent experiments.

### Heparin inhibition and heparinase I treatment

For competition experiments, virus samples were diluted to 100–200 PFU/200 µl in DMEM, and mixed with heparin (Kabi vitrum, Sweden) at different concentrations. Prior to infection, the mixture was incubated for 1 h at 37°C. Subsequently a plaque assay was performed as described above. For the enzyme digestions, BHK-21 cell monolayers at 90% confluence were incubated with different concentrations of heparinase I (Sigma-Aldrich, Germany) for 1 h at 37°C, followed by three washes with PBS prior to infection with 100–200 PFU/200 µl virus samples. The number and size of plaques were then analyzed and recorded.

### Binding of peptides with heparin

Three peptides of E2 glycoprotein from YN87448 and XJ-160 were synthesized by *Beijing Biosynthesis Biotechnology* (China). They were PEP1 (aa 129–150 of YN87448): KIKPKFVGREKYDLPPVHGKKI; PEP2 (aa 129–150 of XJ-160): KIKPKFVGRERYDLPPVYGKNI; and PEP3 (aa 137–169 of YN87448): REKYDLPPVHGKKIPCTVYDRLKE). Radioisotopically labeled heparin and its fragments were prepared by deamination cleavage of heparin [39], followed by reductive labeling with NaB_3_H_4_. The resulting oligosaccharides were separated by gel chromatography and size-defined fractions were collected. Interactions between the peptides and radiolabeled heparin were assessed by nitrocellulose-filter trapping method as described [40]. Briefly, the peptides (5 µg) dissolved in PBS were incubated with 10,000 cpm radiolabeled heparin or its fragments with a polymer degree of 2–24 sugar units in 200 µl PBS (pH 7.4) containing 0.1 mg/ml bovine serum albumin at room temperature for 2 h. The incubated mixtures were rapidly passed through a PBS-washed nitrocellulose filter (diameter 25 mm, pore size 0.45 µm; Sartorius), using a vacuum-assisted filtering apparatus, followed by two washes with 5 ml of PBS. The heparin bound to the membrane together with the peptide was released from the filter with 2 M NaCl, and the radioactivity associated with them was measured by scintillation counting.

### Assay of neurovirulence in suckling mice

Each of the 3-day-old suckling mice (11 in each group) was inoculated intracranially (i.c.) in the right cerebral hemisphere with 30 µl of 10^3^ PFU/ml viruses, respectively. Control mice received an equal volume of Eagle's with 1% FBS injected at the same way. The numbers of dead mice and the time of death were recorded for 72 h postinoculation (p.i.).
